# Supplementary material for: Photocatalytic Activity of TiO2 for the Degradation of Anticancer Drugs
Source: Nanomaterials (Basel). 2022 Oct 9;12(19):3532. doi: 10.3390/nano12193532 (PMC9565840; doi:10.3390/nano12193532)
Supplement: Supplementary file 1 [file nanomaterials-12-03532-s001.zip › nanomaterials-1955780-supplementary.pdf]

Supplementary informations

Photocatalytic activity of TiO_2 for degradation of anticancer drugs

Kristina Tolić Čop[1], Dragana Mutavdžić Pavlović[1*], Tatjana Gazivoda Kraljević[2]

[1] Department of Analytical Chemistry, Faculty of Chemical Engineering and Technology, University of Zagreb, Marulićev trg 19, 10000 Zagreb, Croatia
[2] Department of Organic Chemistry, Faculty of Chemical Engineering and Technology, University of Zagreb, Marulićev trg 19, 10000 Zagreb, Croatia

List of figures:

Figure S1 Nitrates influence on degradation of pharmaceuticals without photocatalyst addition

Figure S2 Absorption spectrum of humic acids and pharmaceuticals

List of tables:

Table S1 MS/MS analysis of IMT

Table S2 MS/MS analysis of CRZ

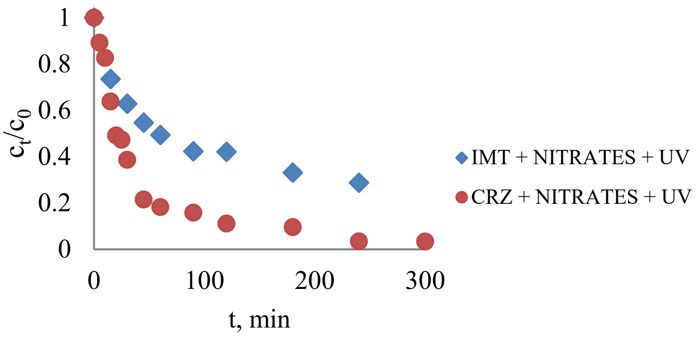

Figure S1 Nitrates influence on degradation of pharmaceuticals without photocatalyst addition

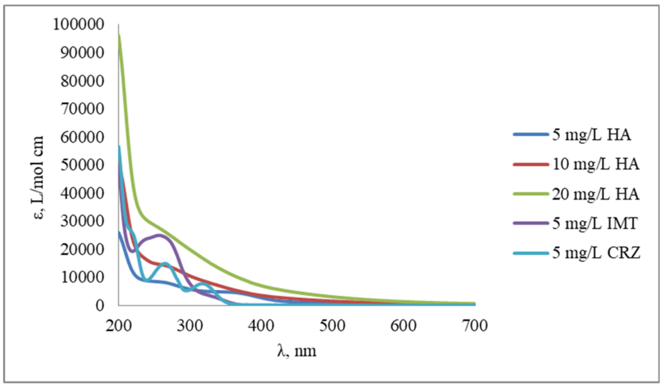

Figure S2 Absorption spectrum of humic acids and pharmaceuticals

Table S1 MS/MS analysis of IMT

Compound	t_R, min	Fragmentation	
		Product ions	collision energy, eV
IMT	5.823	394, 217, 99	20
DP-1	0.866	156, 132,78	20
DP-2	0.900	134, 106, 99	20
DP-3	6.239	478, 423, 394, 277	40
DP-4	7.070	394, 352, 247	40
DP-5	7.170	394, 259, 135	40
DP-6	7.702	408, 380, 261, 149	20
DP-7	8.334	392, 261, 133	40

Table S2 MS/MS analysis of CRZ

Compound	t_R, min	Fragmentation	
		Product ions	collision energy, eV
CRZ	7.013	367, 260, 177	20
DP-1	3.693	272, 229, 189	20
DP-2	3.823	331, 316, 259	40
DP-3	4.637	322, 227, 209	20
DP-4	4.757	414, 387, 304, 253	20
DP-5	6.52	399, 331, 316	40
DP-6	8.147	447, 382, 273	40
DP-7	8.546	381, 356, 285	40
DP-8	8.815	348, 305, 234	40
DP-9	8.915	354, 190	40
DP-10	9.062	288, 177, 112	20
DP-11	9.48	492, 302, 177	20
DP-12	9.644	462, 299, 272	20
DP-13	5.576	219, 202, 190	20
DP-14	7.166	428, 279, 208	40
DP-15	3.387	165, 147, 125	20
